# Structural plasticity in root-fungal symbioses: diverse interactions lead to improved plant fitness

**DOI:** 10.7717/peerj.6030

**Published:** 2018-12-04

**Authors:** Khalil Kariman, Susan Jane Barker, Mark Tibbett

**Affiliations:** 1 School of Agriculture and Environment, The University of Western Australia, Crawley, Western Australia, Australia; 2 Centre for Agri-Environmental Research & Soil Research Centre, School of Agriculture Policy and Development, University of Reading, Berkshire, United Kingdom

**Keywords:** Mycorrhiza, Endophyte, Symbiosis, Root colonization, Interface structures

## Abstract

Root-fungal symbioses such as mycorrhizas and endophytes are key components of terrestrial ecosystems. Diverse in trophy habits (obligate, facultative or hemi-biotrophs) and symbiotic relations (from mutualism to parasitism), these associations also show great variability in their root colonization and nutritional strategies. Specialized interface structures such as arbuscules and Hartig nets are formed by certain associations while others are restricted to non-specialized intercellular or intracellular hyphae in roots. In either case, there are documented examples of active nutrient exchange, reinforcing the fact that specialized structures used to define specific mycorrhizal associations are not essential for reciprocal exchange of nutrients and plant growth promotion. In feremycorrhiza (with *Austroboletus occidentalis* and eucalypts), the fungal partner markedly enhances plant growth and nutrient acquisition without colonizing roots, emphasizing that a conventional focus on structural form of associations may have resulted in important functional components of rhizospheres being overlooked. In support of this viewpoint, mycobiome studies using the state-of-the-art DNA sequencing technologies have unearthed much more complexity in root-fungal relationships than those discovered using the traditional morphology-based approaches. In this review, we explore the existing literature and most recent findings surrounding structure, functioning, and ecology of root-fungal symbiosis, which highlight the fact that plant fitness can be altered by taxonomically/ecologically diverse fungal symbionts regardless of root colonization and interface specialization. Furthermore, transition from saprotrophy to biotrophy seems to be a common event that occurs in diverse fungal lineages (consisting of root endophytes, soil saprotrophs, wood decayers etc.), and which may be accompanied by development of specialized interface structures and/or mycorrhiza-like effects on plant growth and nutrition.

## Introduction

Early terrestrial plants moved from aquatic environments to emerged land about 400 million years ago, when the incipient soil was severely deficient in nutrients and organic matter. Early colonizers were bryophytes such as mosses, liverworts and hornworts, and were composed of a few cells with no true roots, leaves or stems (Kenrick & Crane, 1997). Fossil records indicate that lineages of early land plants established symbiosis with arbuscular mycorrhizal (AM) fungi during the Ordovician period (460 mya) (Redecker, Kodner & Graham, 2000), suggesting that AM fungi aided early colonization of nutrient/water-deficient land by plants via enhancing their nutrient and water uptake. As the ancient soil had an extremely limited reserve of organic matter, the central function of AM fungi presumably was to forage soil for inorganic nutrients, reflected in the functional capacity of extant AM associations.

Mycorrhizal and other (non-pathogenic) root-inhabiting fungi (such as endophytes) continue to play a vital role in soil nutrient cycling, remineralization of organic matter, shaping plant and microbial communities and ultimately safeguarding the stability and functionality of the entire land ecosystem (Jumpponen & Trappe, 1998; Van der Heijden, Bardgett & Straalen, 2008; Weiss et al., 2011; Zuccaro, Lahrmann & Langen, 2014).

Fungal partners can be obligate biotrophs i.e., entirely dependent on living root cells as in AM symbiosis, in which cell architecture and physiology is intensively reorganized to arrange the biotrophic interface (Genre et al., 2005). Some fungal symbionts are facultative biotrophs or partly dependent on their living host such as some ECM fungi or dark septate endophytes (DSE) (Jumpponen & Trappe, 1998; Koide et al., 2008). Sebacinalean endophytes (SE) have both biotrophic and necrotrophic (preceded by pre-programmed death of cortical cells) habits during their interaction with plant roots (Weiss et al., 2011; Lahrmann & Zuccaro, 2012). In return for the assorted benefits conferred to the host plant, the root-associated fungi may depend on their host as the carbon (C) source. Simple sugars (hexoses) were presumed to be the sole C type received by fungal partners in mycorrhizal associations (Smith & Read, 2008), perhaps due to a primary research focus on carbohydrates as a possible food source rather than other organic molecules such as fatty acids (FAs) that were not technically feasible to detect at the time. However, Keymer et al. (2017) fed plants with [U-^13^C6] glucose and monitored the ^13^C patterns of fungal FAs in *Lotus japonicus* wild-types and mutants defective in AM-specific paralogs of lipid biosynthesis genes (*KASI* and *GPAT6*), and demonstrated that plants transfer 16:0 FAs to AM fungi. Accordingly, sugars are now known not to be the only C form provided by host plants in mycorrhizal symbiosis.

Fungal trophic habits are associated with presence/absence of genes involved in degradation of plant cell wall components. Phylogenetic analyses have provided evidence for genomic modifications in certain root fungal symbionts in order to switch from saprotrophic/pathogenic lifestyles towards symbiotic lifestyles (Kohler et al., 2015; Hacquard et al., 2016; Weiss et al., 2016). Convergent loss of saprotrophic traits such as genes encoding plant cell wall degrading enzymes has been documented in ECM fungi (Kohler et al., 2015; Fesel & Zuccaro, 2016), which is a genomic signature for ECM evolutionary transition via saprotrophy to biotrophy, but not in root endophytes such as *Colletotrichum tofieldiae and Serendipita indica* (Lahrmann et al., 2015; Hacquard et al., 2016).

**Figure 1:**
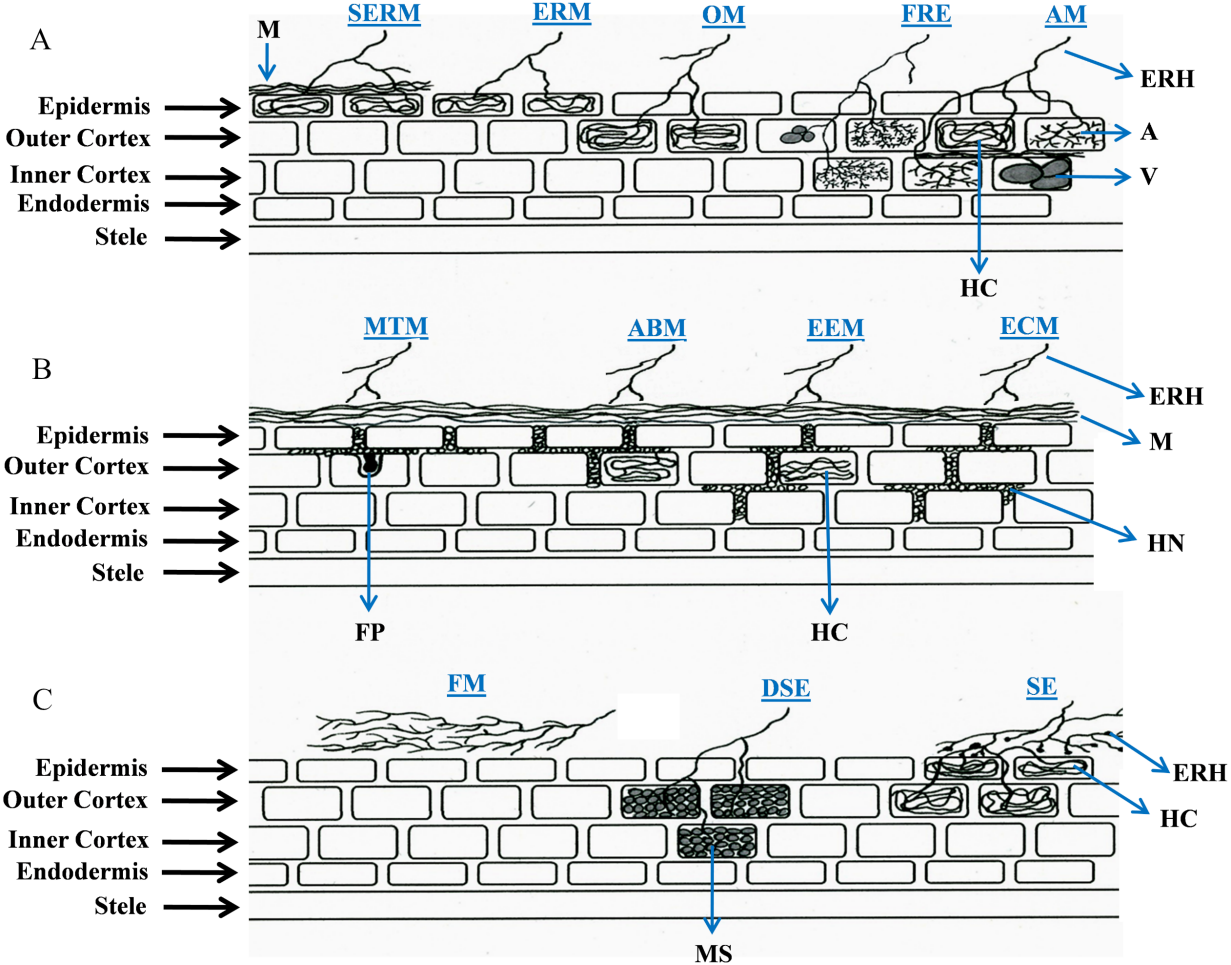
Schematic representation of root colonization strategies in plant-fungal symbioses. The diagram illustrates the interface structures, root cell penetration features of hyphae (i.e., intercellular or intracellular), and also indicates to what extent (epidermis, outer cortex or inner cortex) fungal structures develop. In feremycorrhiza, however, root colonization does not occur. Abbreviations for the symbioses (in blue) are as follows: AM, arbuscular mycorrhiza; ABM, arbutoid mycorrhiza; DSE, dark septate endophytes; ECM, ectomycorrhiza; EEM, ectendomycorrhiza; ERM, ericoid mycorrhiza; FRE, fine root endophytes; FM, feremycorrhiza; MTM, monotropoid mycorrhiza; OM, orchid mycorrhiza; SE, sebacinalean endophytes; and SERM, sheathed ericoid mycorrhiza. Abbreviations for fungal structures (in black): A, arbuscules; ERH, extraradical hyphae; FP, fungal peg; HC, hyphal coils; HN, Hartig net; M, mantle; MS, microsclerotia; and V, vesicles.

Interface structures have been considered to be the defining features of root-fungal interactions, however the underlying mechanisms of nutrient exchange vary across different associations. Interface structures can be specialized (arbuscules, Hartig nets and fungal pegs), non-specialized (hyphae and hyphal coils), or totally absent. In this review, we examine a range of symbiotic relationships between soil-inhabiting fungi and plant roots, focusing primarily on root colonization and interface structures, to address the question of whether the focus on interface structures has limited our understanding of the wider diversity of these symbioses. To date, the types of root-fungal symbioses have been defined by the species involved and the structures developed; namely AM, ECM, ectendomycorrhiza (EEM), arbutoid mycorrhiza (ABM), monotropoid mycorrhiza (MTM), orchid mycorrhiza (OM), ERM (Smith & Read, 2008), sheathed ericoid mycorrhiza (SERM) (Vohnik et al., 2012), SE (Weiss et al., 2011; Zuccaro, Lahrmann & Langen, 2014), DSE (Jumpponen & Trappe, 1998; Koide et al., 2008), fine root endophytes (FRE) (Crush, 1973; Gianinazzi-Pearson et al., 1981; Orchard et al., 2017), fire-associated mutualism (FAM) (Baynes et al., 2011), and a non-colonizing symbiosis (hereafter named “feremycorrhiza”, FM) described recently (Kariman et al., 2014a). Figure 1 illustrates the root colonization features in currently described plant-fungal associations, and Tables 1 and 2 summarize their main characteristics and benefits for host plants, respectively.

**Table 1:** Interface structures in predominantly beneficial root-fungal associations categorized by symbiosis type and partner taxa.

Interface structures in predominantly beneficial root-fungal associations categorized by symbiosis type and partner taxa. Symbiosis	**Plant Taxa**	**Fungal Taxa**	**Interface Structures**	**Intracellular Hyphae**	**Fungal Sheath**
Arbuscular Mycorrhiza (AM) Bryophytes, Pteridophytes, Angiosperms, Gymnosperms	Mucoromycota: Glomeromycotina	Arbuscules	+	+	−
Fine Root Endophytes (FRE)	Angiosperms^b^	Mucoromycota: Mucoromycotina	Arbuscules	+	−
Ectomycorrhiza (ECM)	Angiosperms, Gymnosperms	Basidiomycota, Ascomycota, Mucoromycota	Hartig net	−	+
Ectendomycorrhiza (EEM)	Angiosperms, Gymnosperms	Basidiomycota, Ascomycota, Mucoromycota	Hartig net & Hyphal coils	+	±
Arbutoid Mycorrhiza (ABM)	Ericales: *Ericaceae, Pyrolaceae*	Basidiomycota	Hartig net & Hyphal coils	+	±
Monotropoid Mycorrhiza (MTM)	Ericales: *Ericaceae*	Basidiomycota	Hartig net & Fungal peg	−	+
Orchid mycorrhiza (OM)	Asparagales: *Orchidaceae*	Basidiomycota Ascomycota	Hyphal coils (Pelotons)	+	−
Ericoid Mycorrhiza (ERM)	Ericales: *Ericaceae*	Ascomycota Basidiomycota: Sebacinales	Hyphal coils	+	−
Sheathed Ericoid Mycorrhiza (SERM)	Ericales: *Ericaceae*	Basidiomycota	Hyphal coils	+	+
Sebacinalean Endophytes (SE)	Bryophytes, Pteridophytes, Angiosperms, Gymnosperms	Basidiomycota	Hyphal coils	+	−
Dark Septate Endophytes (DSE)	Angiosperms, Gymnosperms,	Ascomycota	Hyphae & Microsclerotia	+	−
Fire-Associated Mutualism (FAM)^a^	Angiosperms:^b^*Poaceae*	Ascomycota	Unknown	Unknown	−
Feremycorrhiza (FM)	Angiosperms:^b^*Myrtaceae*	Basidiomycota	Absent	No root colonization	−

**Notes.**

a Root colonization features need to be clarified.

b The host plant range needs to be investigated.

**Table 2:** Key benefits of different root-fungal symbioses for host plants.

Symbiosis	Benefits for host	Reference
ABM: Arbutoid Mycorrhiza	^a^Presumably, similar benefits as the ECM symbiosis	Münzenberger, Kottke & Oberwinkler (1992)
AM: Arbuscular Mycorrhiza	Improved mineral nutrition (P and Zn, in particular); tolerance against biotic (pathogens) and abiotic stresses such as drought, salinity, and heavy metals; improved soil health and structure	Smith & Read (2008), Sikes (2010), Hildebrandt, Regvar & Bothe (2007)
DSE: Dark Septate Endophytes	Breaking down various organic substances and releasing nutrients; protection against plant pathogens, herbivores, and abiotic stresses such as heat, presumably due to the fungal capability of synthesizing antibacterial and antifungal compounds, toxic secondary metabolites, or high melanin contents of hyphae	Jumpponen & Trappe (1998), Newsham (1999), Newsham (2011)
ECM: Ectomycorrhiza	Improved plant vigour and access to nutrients that are tightly fixed in complex organic matter or soil particles (N and P in particular); alleviating stresses caused by soil-borne pathogens and abiotic stresses such as drought and salinity; ameliorating the CO_2_ fertilization effect; accelerating weathering of rocks and releasing essential nutrients; decomposition of soil organic matter, tolerance to P toxicity	Landeweert et al. (2001), Smith & Read (2008), Terrer et al. (2016), Kariman et al. (2014b)
EEM: Ectendomycorrhiza	Hydrolyzing complex polysaccharides and supplying C to young host seedlings prior to the beginning of their autotrophism; possibly involved in revegetation of disturbed sites and establishment of conifer seedlings post-fire	Trevor et al. (2001); Navarro-Ródenas et al. (2012)
ERM: Ericoid Mycorrhiza	Assisting plants to survive in nutrient impoverished habitats through mineralization and acquisition of nutrients from soil organic sources	Read, Leake & Perez-Moreno (2004)
FAM: Fire-Associated Mutualism	Thermotolerance and fire adaptation by enhancing both probability of fire (via increased plant biomass) and plant survival (larger underground seed bank)	Baynes et al. (2011)
FM: Feremycorrhiza	Improved plant growth and nutrition mainly via increasing nutrient solubilization and mobilization; tolerance to P toxicity	Kariman et al. (2014a), Kariman et al. (2014b)
FRE: Fine Root Endophytes	Possibly involved in tolerance against extreme environmental conditions such as high altitude, soil acidity, cold temperatures, and waterlogging. Functional traits are not well known	Postma, Olsson & Falkengren-Grerup (2007), Orchard et al. (2016)
MTM: Monotropoid Mycorrhiza	Supplying C (sourced from neighbouring trees) to mycoheterotrophic host plants	Tedersoo et al. (2007), Hynson et al. (2013)
OM: Orchid mycorrhiza	Feeding host plants during their mycoheterotrophic phase, mainly through breaking down simple/complex organic matter	Cameron, Leake & Read (2006)
SE: Sebacinalean Endophytes	Improved growth and resistance against biotic and abiotic stresses such as salinity	Varma et al. (2001), Weiss et al. (2011), Weiss et al. (2016)
SERM: Sheathed Ericoid Mycorrhiza	^a^Presumably, similar benefits as the ERM symbiosis	Vohnik et al. (2012)

**Notes.**

a There is limited experimental evidence about the potential benefits for host plants.

The location of the plant-fungal interaction on the mutualism–parasitism spectrum ultimately depends on the nutritional balance achieved by the partnership rather than the type of interface structure that is formed (Smith & Smith, 1996). Regardless of the symbiosis type and morphological traits, the net outcome of root-fungal interactions for host plants (positive, neutral, or negative responses) is determined by environmental conditions (e.g., nutrient availability in soil), plant/fungus genetics, and temporal relationships during mycorrhizal development (Johnson, Graham & Smith, 1997; Klironomos, 2003).

Three waves of mycorrhizal evolution have been proposed by Brundrett & Tedersoo (2018) including (i) the origin of AM associations in early terrestrial plants over 450 Mya, (ii) evolution of ECM (except *Pinaceae*), nonmycorrhizal (NM) roots, ERM, and OM associations along the period of major root diversification in the Late Cretaceous, and iii) development of specialized nutrition strategies and multifunctional (e.g., AM and ECM) roots in a few plant families. The third wave is an ongoing trend that is related to substantial climate change since the Palaeocene. Recent studies have revealed that the actual niches of different root symbionts could extend beyond those our respective domains of research predict (Selosse, Schneider-Maunoury & Martos, 2018). Evolutionary tendency towards shifting ecological niches (evolutionary trajectories) seems to be a common event in fungi that occurs convergently in numerous independent taxa. In fungal root symbionts, the key evolutionary trajectories include (i) additional food source plus protection against soil adversities and (ii) regressive evolution, such as that of enzymes involved in saprotrophic nutrition (Selosse, Schneider-Maunoury & Martos, 2018). Transitions between nutritional strategies in root-fungal relationships, especially gains or losses of ECM and NM strategies, are more common than previously thought (Brundrett & Tedersoo, 2018). In this review, we have briefly mentioned the ecological niches of certain root symbionts, which seem to have been overlooked.

The majority of plants may be simultaneously associated with a multitude of symbiotic fungal clades in their natural ecosystems. Mycobiome studies using the state-of-the-art “omics” technologies have unearthed a range of root endophytic and root-colonizing saprotrophic relationships involving diverse fungal lineages in both mycorrhizal and NM plants (Shakya et al., 2013; Toju et al., 2013; Oliveira et al., 2014; Toju, Sato & Tanabe, 2014; Coleman-Derr et al., 2016; Glynou et al., 2016; Almario et al., 2017). For example, two independent studies using 454-pyrosequencing have revealed diverse mycorrhizal and endophytic taxa associated with roots of 36 co-occurring plant species in an oak-dominated forest in northern Japan (Toju, Sato & Tanabe, 2014), and three endangered orchid species in the Atlantic Forest, in Brazil (Oliveira et al., 2014). These studies support the proposition that beneficial root-fungal interactions could extend far beyond the well-established associations mentioned above (Fig. 1). It is increasingly apparent that study of plant performance in the absence of the co-evolved rhizosphere biota will give a limited and even skewed understanding of plant functioning in their natural environments (Smith et al., 2011) e.g., for applications such as increasing sustainability of food production in low input agroecosystems, and habitat restoration for recovery of endangered species (Philippot et al., 2013). Efforts such as those to ameliorate degraded lands may be further hampered by insufficient understanding of the complex variety of plant-fungal interactions that may also need to be restored (Bever et al., 2010).

Overemphasizing the visibility of symbioses (i.e., root colonization and interface specialization) could have hindered our understanding of the actual functional contribution of root-associated fungi towards health and performance of terrestrial ecosystems. This review examines the morphological (interfacial matrix) and functional (primary focus on plant fitness) aspects of diverse symbioses with the aim of engendering discussion about a broader view of plant-fungal root symbioses that emphasizes both form and function. Furthermore, our modest knowledge of fungal taxonomy and ecology also contributes to our limited understanding of the functioning of root fungal symbionts, hence we will explore the taxonomically/ecologically diverse interactions observed recently by aid of high-throughput “omics” technologies.

### Current definition of symbiosis

It is necessary to mention the current definition of symbiosis, which is arguably the most controversial term in the history of biological terminology (Martin & Schwab, 2013). The term “symbiotismus” was coined by Albert Bernhard Frank in 1877 to describe the mutualistic relationship in lichens, whereas the word “symbiosis” is credited to Anton de Bary who, one year later, defined symbiosis as “the living together of unlike named organisms” (Sapp, 1994; Sapp, Carrapiço & Zolotonodov, 2002). While some scientists persisted in the opinion that “symbiosis” should only be used for mutualistic relationships, others extended the definition to encompass commensalistic and parasitic interactions (Douglas, 2010). The 130-year-old debate is over now and the current general biology and ecology textbooks use the broader definition of symbiosis that includes all possible interaction types, and the restrictive definition (equalizing symbiosis with mutualism) has essentially disappeared (Martin & Schwab, 2013). Symbiosis (which literally means “living together”, derived from the Greek word “sym”: together, and “biosis”: living) is currently defined as any type of close and long-term ecological relationship between two living organisms of different species. Here, we explore the root-fungal symbioses that are predominantly beneficial to host plants i.e., mutualistic and commensalistic symbioses, where the fungal partner may or may not benefit from the association, respectively.

### Interfacial and functional properties of root-fungal symbioses

#### Arbuscular mycorrhiza (AM)

Approximately 80% of all vascular plant species establish AM symbiosis (72% are AM consistently, while 7% form inconsistent NM-AM associations) including bryophytes, pteridophytes, gymnosperms and angiosperms (Brundrett & Tedersoo, 2018) with the fungi that were, until very recently, classified within the phylum Glomeromycota (Smith & Read, 2008; Schussler & Walker, 2010). Spatafora et al. (2016) conducted extensive phylogenetic analyses of a broad range of fungal taxa and consequently split zygomycetes into two phyla Mucoromycota (mainly mycorrhizas, root endophytes and saprotrophs) and Zoopagomycota (mainly pathogens of small animals and fungi), and placed AM fungi (AMF) within Mucoromycota as a subphylum i.e., Glomeromycotina. Although AMF are considered to be nonspecific or have very low species-level specificity (Smith & Read, 2008), the extent of colonization and AMF composition may vary for different plant-fungus combinations. AMF communities can be influenced by plant identity, ecological groups (e.g., habitat generalist plants vs habitat specialist plants) or the ecosystem type (Gollotte, Van Tuinen & Atkinson, 2004; Öpik et al., 2009; Veresoglou & Rillig, 2014). For example, Vandenkoornhuyse et al. (2003) demonstrated that even coexisting grass species can have significantly different AMF communities. With respect to ecological preferences, specialist AMF taxa were shown to have a tendency towards habitat specialist plants, while generalist AMF prefer habitat generalist plants in both forest and grassland ecosystems (Öpik et al., 2009; Vályi, Rillig & Hempel, 2015).

The main benefits plants receive from AM symbiosis include access to the soil nutrients that are otherwise unavailable and resistance against pathogens and environmental stresses such as drought, salinity and heavy metal toxicity (Smith & Read, 2008). AM fungi are not able to complete their lifecycle in the absence of a host and this effect has not been able to be overcome by tissue culture techniques. The close evolutionary relationship extending over the entire period of plant evolution on land, and the remarkable intracellular development of these fungi that involves very close interaction between the plant and fungal nuclei suggests this symbiosis has evolved towards the same host nucleus dependence that have eukaryote cell organelle symbioses (Barker, Tagu & Delp, 1998), and other specialized microbial symbioses such as legume-rhizobium associations (Coba de la Peña et al., 2017).

Although AM fungi generally colonize roots, colonization of root hairs has also been documented (Guinel & Hirsch, 2000). Hyphae grow inter- and intracellularly through living epidermal and cortical cells, in which they produce specialized shrub-shaped structures called arbuscules. Prior to hyphal penetration, host epidermal cells located immediately below the fungal appressorium undergo intensive re-programming, such as remodeling of actin filaments and microtubules, and form a pre-penetration apparatus (PPA, Genre et al., 2005). Therefore, PPA formation is an appropriate cellular marker indicating which epidermal cells are about to be colonized. The PPA development includes differential expression of genes involved in cell wall modification such as expansin-like gene and cellulose synthase (Siciliano et al., 2007). Following penetration into the root cortex via extracellular or intracellular hyphal growth, and depending on the plant-fungus combination, AM associations may produce arbuscules (Arum-type), thick hyphal coils (Paris-type), or intermediate structures (such as arbusculate coils) that cannot be easily defined (Smith & Smith, 1997; Dickson, 2004; Karandashov et al., 2004). Arbuscules are separated from the cytoplasm by a pre-arbuscular membrane, originated by invagination of plant plasma membrane, and are specialized interface structures where the fungal partner trades nutrients such as P, taken up from soil using its extensive extraradical hyphae, for simple carbohydrates from the host plant (Harrison, 2012). Arbuscules are preferentially formed inside inner cortical cells. Similar to arbuscules, hyphal coils are surrounded by plant plasma membranes. The morphological variability of AM structures may be influenced by plant cultivars, fungal isolates and soil conditions (Dickson, 2004). Arum-type associations may be superior in supplying nutrients to their host due to growing intercellularly in a longitudinal pattern (a feature missing in the Paris-type), leading to higher colonized root length. The Paris-type structures, however, live longer than arbuscules. Although the total percentage of root colonization (counting all intraradical structures) is generally considered to be an indicator of the AM presence and functionality, the extent of colonization does not necessarily reflect the physiological responses of the plant host (Jakobsen, 1995; Smith, Smith & Jakobsen, 2004; Kariman et al., 2014b). This could be related to relative abundance and activity of arbuscules as well as the extent of development of extraradical hyphae and their effectiveness in nutrient uptake.

Recent work highlights the fungal influence on nutrient transporters in roots, which differs for different species interactions (Smith, Smith & Jakobsen, 2004; Smith et al., 2011; Kariman et al., 2014a). Nutrient-related signals seem to control the development of AM symbiosis. Two conserved cis-elements (MYCS and P1BS) in the promoter regions of AM-inducible phosphate transporter genes are required simultaneously for symbiotic gene expression and root colonization (Karandashov et al., 2004; Chen et al., 2011). Coordination of inorganic P (Pi) starvation and AM signalling pathways appears to be the prerequisite for establishment of the symbiosis, but the precise regulatory mechanisms underlying this phenomenon are not known. Although generally considered a beneficial association for host plants, the net effects of AM associations on the host plant can be positive, neutral, or even negative depending on the plant-fungus combination and genetics, origin of the fungal isolates, and environmental context (Klironomos, 2003; Smith, Grace & Smith, 2009).

### Fine Root endophytes (FRE)

Fine endophytes are root endophytic fungi associated with angiosperms that have been traditionally classified as AM fungi (*Glomus tenue*) (Crush, 1973; Gianinazzi-Pearson et al., 1981), but their taxonomic position has been quite ambiguous (Schussler & Walker, 2010) until recently. Orchard et al. (2017) clarified the phylogenetic position of FRE and demonstrated that these fungi belong to Mucoromycotina, not Glomeromycotina. Typical features of FRE include fine hyphae of less than 1.5 µm in diameter, intracellular colonization of epidermal and cortical cells in a fan-like branching pattern, and production of arbuscule- and vesicle-like structures (Gianinazzi-Pearson et al., 1981; Abbott, 1982; Orchard et al., 2017). Capability of Mucoromycotina to establish symbiosis with early terrestrial plants has raised the hypothesis that Mucoromycotina may be predecessors of Glomeromycotina (Bidartondo et al., 2011; Hoysted et al., 2018). The hypothesis is further supported by the close phylogenetic relationship between Mucoromycotina and AM fungi (Lin et al., 2014; Spatafora et al., 2016), recent demonstration of a mutually beneficial symbiosis between Mucoromycotina and Haplomitriopsida liverworts (Field et al., 2015), and their arbuscule-forming feature (Orchard et al., 2017). Improved growth of ryegrass (*Lolium perenne*), cocksfoot (*Dactylis glomerata*), and sweet vernal (*Anthoxanthum odoratum*) associated with FRE has been observed under P-deficient conditions (Crush, 1973). FRE may have important ecological functions in plants exposed to abiotic stresses due to their ubiquitous presence under extreme environmental conditions such as high altitude, soil acidity, cold temperatures, and water-logging (Crush, 1973; Postma, Olsson & Falkengren-Grerup, 2007; Newsham, Upson & Read, 2009; Orchard et al., 2016). The available evidence suggests that the role of FRE fungi in supporting healthy plant communities should be further examined (Orchard et al., 2016).

### Ectomycorrhiza (ECM)

Around 2% of all vascular plant species (∼8,500 species) form ECM symbiosis (Brundrett & Tedersoo, 2018). ECM hosts are mostly woody species belonging to *Betulaceae*, *Fagaceae*, *Dipterocarpaceae*, *Myrtaceae*, *Pinaceae*, *Salicaceae*, *Cupressaceae*, *Rosaceae*, *Fabaceae*, *Aceraceae*, *Euphorbiaceae*, and *Ulmaceae*, whilst there are up to 20,000 basidiomycetous, ascomycetous and Mucoromycotina fungi involved in this symbiosis (Rinaldi, Comandini & Kuyper, 2008; Tedersoo, May & Smith, 2010). Climatic factors have been suggested as the main drivers of ECM diversity and distribution globally, and the species richness of ECM fungi peak at mid-latitudes, especially in temperate forests and Mediterranean biomes of the Northern Hemisphere i.e., where pine forests are the dominant vegetation (Tedersoo et al., 2014; Mello & Balestrini, 2018). Environmental and host factors have been shown to drive ECM diversity across the European forests (Van der Linde et al., 2018).

A sheath of organized hyphae called a mantle is formed around fine lateral roots. The colonized lateral roots have restricted apical growth (referred to as “short roots”), are thicker than non-colonized roots and have no root hairs. Some short roots have bifurcating branching patterns, which is an ontogenetic plant trait (Smith & Read, 2008). Mantle functions as a transitory storage compartment for metabolites received from the host and also the nutrients absorbed from soil by extraradical hyphae (Smith & Read, 2008; Nehls & Bodendiek, 2012). Mantle might be less developed (sparse and loose) in some ECM associations (Walker, 1985). The presence of fifteen genes encoding putative hexose transporters and the absence of genes involved in sucrose hydrolysis (invertases) in the genome of the model ECM fungus *Laccaria bicolor* suggest that this fungus receives its C source as simple six-C sugars such as glucose and fructose (Martin et al., 2008). However, this does not seem to be a general trend. An invertase gene is present in the genome of the ascomycetous ECM fungus *Tuber melanosporum,* making it capable of hydrolyzing sucrose and potentially less dependent on the host plant (Martin et al., 2010). Furthermore, carbohydrates might not be the sole C form provided by host plants, considering the evidence of FAs transfer from host plants to AM fungi (Keymer et al., 2017). From the mantle, extraradical hyphae extend outward into soil, and also intraradical hyphae grow between the root epidermal and cortical cells. The biotrophic interface is called a Hartig net. It is composed of highly branched labyrinthine hyphae that grow between epidermal cells (angiosperms) or penetrate in the epidermis and surround several layers of cortical cells (gymnosperms). The Hartig net is well-known as the active site for nutrient exchange between the two symbiotic partners (Smith & Read, 2008). However, there are reports of unusual ECM associations lacking a Hartig net, where positive growth and nutritional responses have been observed (Brundrett, 2009). ECM symbiosis is crucial for the succession of land ecosystems as it improves plant vigor and access to nutrients that are tightly fixed in complex organic matter or soil particles (N and P in particular), alleviates stresses caused by soil-borne pathogens and abiotic stresses such as drought and salinity, ameliorate the CO_2_ fertilization effect, and can have a role in weathering of rocks and decomposition of organic matter in soil (Landeweert et al., 2001; Tibbett & Sanders, 2002; Smith & Read, 2008; Terrer et al., 2016). Saprotrophy to biotrophy transition seems to be a central trend in evolution of ECM fungi. Multigene phylogenetic analyses have suggested that ECM fungi have evolved independently from their saprotrophic and wood decaying ancestors (James et al., 2006), at least 80 times (Tedersoo & Smith, 2013). Recently, some wood-decaying fungi were shown to colonize the roots of coniferous seedlings in a mycorrhizal manner, some of which formed typical ECM structures (Smith et al., 2017). ECM-forming *Tuber* species have also been observed to colonize the roots of herbaceous plants in an endophytic manner (Gryndler et al., 2014; Schneider-Maunoury et al., 2018). An endophytic interaction has been documented between the ECM fungus *Tricholoma matsutake* and the AM tree *Cedrela odorata* (Murata et al., 2013). These examples support proposals that (i) there is a wider ecological niche for ECM fungi i.e., some also act as a saprotroph/endophyte probably depending on fungal requirement for C and/or (ii) some ECM fungi are at different transitory stages within the saprotrophy-biotrophy continuum.

### Ectendomycorrhiza (EEM)

The EEM association is formed between gymnosperms/angiosperms, including plant species belonging to *Pinus* (pine), *Picea* (spruce) and *Larix* (larch), and certain members of the fungal taxa Ascomycota, Basidiomycota and Mucoromycota (previously within Zygomycota), including species of *Wilcoxina*, *Sphaerosporella*, *Phialophora* and *Chloridium* (Egger & Fortin, 1988; Trevor et al., 2001; Navarro-Ródenas et al., 2012). Hartig net and mantle develop in EEM associations similar to the ECM, and hyphae also penetrate epidermal and cortical cells as observed in AM symbiosis. The mantle is quite thin and sometimes absent. The Hartig net grows distal to the root apical meristem between the epidermal and outer cortical cells and may progress deeper between inner cortical cells resulting in short roots as seen in ECM associations. Intracellular hyphae are highly branched, and usually develop in the vicinity of the cells surrounded by the Hartig net. Dense bodies of polysaccharides (probably glycogen) were detected in the mantle and Hartig net in the *Pinus banksiana*-*Wilcoxina* association (Scales & Peterson, 1991). However, there is no direct evidence for nutrient exchange at the symbiotic interface and like the majority of features of less common symbioses, this potential process needs to be further investigated. EEM fungi can hydrolyze complex polysaccharides, so they have been proposed to be the suppliers of C to young seedlings prior to the beginning of their autotrophism (Egger, 1986). Presence of EEM symbiosis in seedlings regenerating from burned coniferous/deciduous forests suggests that EEM fungi may play a role in revegetation of disturbed sites and establishment of conifer seedlings post-fire (Mikola, 1965; Lobuglio & Wilcox, 1988; Trevor et al., 2001).

### Arbutoid mycorrhiza (ABM)

Plants from *Ericaceae* and *Pyrolaceae*, mostly species of *Comarostaphylis, Arctostaphylos* and *Arbutus*, establish ABM associations with diverse basidiomycetous fungi (Münzenberger, Kottke & Oberwinkler, 1992). ECM-forming fungi may mediate the interactions between ABM and ECM plants as they establish ECM symbiosis with trees, and can also form ABM associations with ericaceous plants from the Arbutoidea suborder (Richard et al., 2005). The mycorrhizal structures are very similar to those of ECM and the fungal species involved are also capable of forming ECM associations, so the plant species often determines the type of the association present in a given area of forest. In these associations, a hyphal mantle covers the root surface but might be missing, and a Hartig net is usually restricted to epidermal and outer cortical cells due to the presence of suberin deposits and Casparian strips in the outer cortex of host roots (Münzenberger, 1991). Furthermore, hyphae also penetrate the outer cortical cells and fill them with coils, which is the distinguishing feature of ABM as compared to ECM symbiosis (Münzenberger, Kottke & Oberwinkler, 1992) and is reminiscent of the Arum-Paris division of AM symbioses that is also host-driven (Smith & Smith, 1997). Although the ABM symbiosis is presumed to be functionally similar to ECM, there is very limited knowledge about the exchange of nutrients and possible benefits for each partner.

### Monotropoid mycorrhiza (MTM)

Achlorophyllous plants from *Ericaceae*, such as *Monotropa*, *Pterospora* and *Sarcodes* establish MTM symbiosis with several basidiomycetous fungi (that also form ECM symbioses with neighboring photosynthetic plants) including species of *Boletus, Lactarius*, *Rhizopogon*, *Russula*, and *Tricholoma* (Cullings & Bruns, 1996; Bidartondo & Bruns, 2002; Bidartondo, 2005; Yang & Pfister, 2006). A dense fungal sheath is formed around roots that may or may not enclose the root apex, depending on the plant species involved. A Hartig net develops but is confined to the outer epidermal cells and does not extend deeper into cortex presumably due to presence of cell wall modifications such as Casparian strips (Bonfante & Perotto, 1995). Individual hyphae from the Hartig net or inner mantle grow into epidermal cells but do not penetrate cell walls i.e., cell walls bend to accommodate the penetrated hyphae, a structure designated as a “fungal peg”. This results in increased surface area of the cell, which can potentially facilitate nutrient transfer at the interfacial matrix although there is no direct contact with the plant cytoplasm. Fungal pegs are presumed to be the active site for nutrient exchange. There is no direct evidence in this regard but the development of additional structures supports this proposal. A membranous sac is extended from the fungal peg into the host cytoplasm and, small wall ingrowths (protuberances) grow from the invaginated cell walls into epidermal cells (Hynson et al., 2013). Presence of a fungal peg and lack of intracellular hyphal structures are the main features distinguishing MTM from ABM and as the same fungi can produce both types of association (below), this is another example where symbiotic structures are directed by the host.

In MTM associations, the host plants contain no chlorophyll and are mycoheterotrophic i.e., they rely on their fungal partners for the C to be sourced through neighboring autotrophic plants such as *Pinus*, *Picea*, *Fagus*, *Quercus*, *Cedrus*, and *Salix* (Leake, 1994; Tedersoo et al., 2007; Hynson et al., 2013). Mycoheterotrophic plants are considered as the only unambiguous examples for the potentially wide-spread phenomenon of plant-to-plant C transfer via hyphal networks of mycorrhizal fungi (Bidartondo, 2005). Glucose molecules labeled with ^14^C radioisotope were injected into the phloem of pine and spruce trees, and were subsequently detected in the mycoheterotrophic *Monotropa* plants within five days (Bjorkman, 1960), demonstrating that the MTM host receives its C from the neighboring plants through the common fungal hyphae. The saprotrophic capacity of the fungal symbiont, therefore, seems very unlikely and it is not also clear whether the plant partner supplies any types of C to the fungus or it comes entirely from the neighboring trees. The relationship between the host plant and the neighboring trees could be of a reciprocal nature as ^32^P injected into flowering stems of the MTM host *Monotropa uniflora* was detected in the neighboring *Quercus* trees (Furman, 1966).

### Orchid mycorrhiza (OM)

Orchids from the plant family *Orchidaceae,* that encompasses 10% of vascular plant species, establish OM symbiosis (Brundrett & Tedersoo, 2018), with a variety of basidiomycete (including *Ceratobasidium, Russula*, *Sebacina* and *Tulasnella*), and ascomycete (including *Tuber, Peziza* and *Tricharina* species) fungi (Selosse et al., 2004; Smith & Read, 2008; Waterman et al., 2011). Fungal hyphae colonize newly formed root hairs soon after seed germination. Hyphae penetrate cells and become surrounded by the invaginated plasma membrane. Coarse hyphal coils (pelotons) are formed within cells and dramatically increase the interfacial surface between the two partners (Rasmussen, 2002; Smith & Read, 2008). Similar to arbuscules, pelotons have short life-spans of a few days, then are degenerated and digested by the host cells, which remain functional and can be recolonized by other hyphae (Rasmussen, 2002).

Unlike most plants, all orchids have a mycoheterotrophic stage at some point during their life cycle. Orchid seeds have a very limited reserve of nutrients, so all orchids are completely reliant on their mycorrhizal partners during their early growth stage, which is called a protocorm (Smith & Read, 2008; McCormick et al., 2012). Similar to what we may expect in other plant-fungal interactions, three possible outcomes from the initial contact between the fungus and imbibed seeds are: formation of functional mycorrhiza; seed parasitism; rejection of fungal colonization. Only seeds involved in functional mycorrhizal interactions successfully germinate and grow. After the protocorm stage, the majority of the adult orchids become autotrophic and putatively independent of C supply from the fungal partners, however, some orchids (mycoheterotrophic species) remain totally dependent on the mycorrhizal fungus to gain C and nutrients such as phosphate and N (Alexander, Alexander & Hadley, 1984; Cameron, Leake & Read, 2006; Rasmussen & Rasmussen, 2009). As in most mycorrhizal associations, the plant and fungal supply of organic and mineral nutrients can originate from the fungal capability to utilize simple and complex carbohydrates such as glucose, mannose, cellobiose, cellulose, CMC, xylan, pectin and starch, and also inorganic/organic sources of N (ammonium, amino acids and proteins) and P (orthophosphate, DNA and phytic acid) (Nurfadilah et al., 2013). Studies by Cameron, Leake & Read (2006) showed that considerable quantities of fixed C in green forest orchid *(Goodyera repens)* were allocated to the extraradical mycelium of the OM fungi involved, demonstrating mutualism in OM symbiosis. Taken together, OM fungi can acquire their C source through either their heterotrophic activities or the autotrophic partner, and the extent of contribution of each route could be context-dependent.

Some mycoheterotrophic orchids are not fungal-specific, and can be associated with diverse saprophytic fungi. For instance, an OM association between the Asiatic *Epipogium roseum* and the saprotrophic *Psathyrellaceae* was successfully established and developed (Yamato et al., 2005; Yagame et al., 2007). Furthermore, diverse saprotrophic lineages have been identified in orchids including *Psathyrellaceae* (Ogura-Tsujita & Yukawa, 2008), *Mycenaceae* (Ogura-Tsujita et al., 2009; Selosse et al., 2010), *Marasmiaceae* (Dearnaley & Bougoure, 2010), *Resinicium* spp (Martos et al., 2009), and *Gymnopus*-related fungi (Dearnaley, 2006). These studies indicate less specificity in OM associations, and further highlight the fact that mycorrhizal fungi can be quite versatile in terms of their nutritional lifestyles, and may behave as saprotrophs or biotrophs.

### Ericoid mycorrhiza (ERM)

Plants from *Ericaceae* (1.5% of vascular plant species) such as *Calluna* (heather), *Vaccinium* (bilberry) and *Erica* (heath) form ERM symbiosis with various ascomycetous fungi including *Rhizoscyphus ericae*, *Meliniomyces variabilis*, and *Oidiodendron maius* (Rice & Currah, 2006; Brundrett & Tedersoo, 2018). Although many sebacinalean taxa have also been detected in ERM roots in DNA-based molecular studies (Bruzone, Fontenla & Vohník, 2015), it is still unclear whether they form true ERM associations or they are common mycobionts or endophytes associated with roots. ERM fungi may form a loose hyphal network around the fine hair root surfaces. They penetrate the epidermal/cortical cells (without penetrating plasma membrane) and fill them with dense hyphal coils. ERM colonization is restricted to expanded (mature) cells, so the root apical region remains uncolonized. Recently, a new symbiosis with distinct morphological characteristics was discovered between *Vaccinium* spp. and an undescribed non-sebacinalean basidiomycete, termed “sheathed ericoid mycorrhiza” (SERM) (Vohnik et al., 2012). In this symbiosis, fungal hyphae produce 1- to 3-layer sheaths around the terminal parts of hair roots and fill rhizodermal cells with hyphal coils.

Reciprocal exchange of C and P has been demonstrated in an ERM association between *R. ericae* and *Calluna vulgaris* (Pearson & Read, 1973)*.* Similarly, a reciprocal exchange of C and P has been observed between the ERM fungus *Pezoloma ericae* and the leafy liverwort *Cephalozia bicuspidata* (Kowal et al., 2018). ERM fungi enhance fitness and nutrient acquisition of *Ericaceae* plants in their natural habitats through decomposition of complex organic compounds by secreting a broad range of enzymes including cellulases, proteases, polyphenol oxidases and phosphatases (Read, Leake & Perez-Moreno, 2004; Martino et al., 2018). They also protect host plants against heavy metal toxicity (Perotto, Girlanda & Martino, 2002) and may have a role in the restoration of ericaceous heaths (Diaz et al., 2006; Diaz, Green & Tibbett, 2008).

ERM fungi play a vital role in survival of ERM plants growing in nutrient impoverished habitats (typically having low soil pH and slow turnover of organic matter), through mineralization of soil organic matter. Martino et al. (2018) compared the genomes of four ERM fungi (*Meliniomyces bicolor*, *Meliniomyces variabilis*, *Oidiodendron maius* and *Rhizoscyphus ericae*) with those of fungi with different lifestyles (mycorrhizas, endophytes, saprotrophs, and pathogens). The ERM fungal genes related to saprotrophy (such as those encoding polysaccharide-degrading enzymes, lipases, and proteases) and secondary metabolism were shown to be closer to those of saprotrophs and pathogens than those found in the ECM fungi. The most highly upregulated genes in the ERM symbiosis (biotrophic interaction) were those encoding fungal and plant cell wall-degrading enzymes (CWDEs), lipases, proteases, nutrient transporters and mycorrhiza-induced small secreted proteins (MiSSPs). Accordingly, as reflected in the ERM fungal gene repertoire, conservation of a dual lifestyle capacity (saprotrophic and biotrophic) in ERM fungi seems to be a necessity for mutual plant-fungus fitness.

### Sebacinalean endophytes (SE)

SE fungi, such as *Serendipita indica*, belong to Sebacinales (Hymenomycetes, Basidiomycota). These fungi colonize a broad range of bryophytes, pteridophytes, angiosperms (monocots and dicots) and gymnosperms, including the non-mycorrhizal plant *Arabidopsis thaliana*, without causing disease symptoms (Varma et al., 2001; Weiss et al., 2011; Lahrmann et al., 2013). A loose hyphal network is formed around the roots and hyphae grow inter- and intracellularly in the epidermis and also the outer layer of cortex in some plant species, but do not colonize the root tips and meristematic zones (Deshmukh et al., 2006; Zuccaro et al., 2011). Morphologically-distinct interface structures have not been detected at the symbiotic interface.

Host plants gain various benefits from the SE partners including improved growth and resistance against various biotic and abiotic stresses such as salinity (Waller et al., 2005; Deshmukh et al., 2006; Weiss et al., 2011). Bi-directional exchange of nutrients/C has been observed between SE fungi and their hosts (Yadav et al., 2010; Zuccaro et al., 2011; Zuccaro, Lahrmann & Langen, 2014), however a specialized means of nutrient transfer has not been reported. Within cells, hyphae are enveloped by host plasma membrane and establish a biotrophic interaction. They also cause pre-programmed cell death of cortical cells, in which they subsequently degrade organic compounds in a saprotrophic manner via secretion of extracellular enzymes such as proteases and metalloproteases (Zuccaro et al., 2011; Lahrmann & Zuccaro, 2012; Zuccaro, Lahrmann & Langen, 2014). Accordingly, the fungal symbiont is known to possess a dual lifestyle involving biotrophic and hemi-biotrophic (necrotrophic) phases. Sebacinales is an important fungal order for evolutionary studies of root symbionts, consisting of lineages that have highly diverse interactions with plants including saprotrophism, endophytism, and mycorrhizal associations (Weiss et al., 2016). In contrast to facultative and obligate biotrophic symbionts, genes associated with saprotrophy and necrotrophy habits are well represented in the genome of the hemibiotrophic endophyte *S. indica* including members of the glycoside hydrolase families, metallo-endopeptidase families, and caspase family (Kohler et al., 2015; Lahrmann et al., 2015; Van der Heijden et al., 2015). However, genes that are strictly involved in lignin degradation such as those encoding class II peroxidases (PODs) are absent from the genome of *S. indica*, similar to most ECM fungi (Kohler et al., 2015). This might underly loss of the wood-decaying capacity of these fungi during evolution towards the biotrophy/hemibiotrophy traits.

### Dark septate endophytes (DSE)

DSE are polyphyletic aggregates of fungi belonging to Ascomycota, conidial or sterile, and share melanized and septate hyphae as their common morphological traits (Rodriguez & White, 2009). *Acephala applanata*, *A. macrosclerotiorum*, *Phialocephala fortinii*, *P. glacialis Vibrissea truncorum*, and *Meliniomyces variabilis* are among the ascomycetous fungi forming DSE associations (Grunig, Queloz & Sieber, 2011). They colonize roots from a variety (nearly 600 species) of angiosperms and gymnosperms without causing any apparent disease symptoms (Jumpponen & Trappe, 1998). Hyphae grow in roots inter- and intracellularly and like SE do not form specialized interface structures for nutrient exchange. There are four distinct fungal structures formed by DSE (Jumpponen & Trappe, 1998). Runner hyphae are individual hyphal strands linked to depressions between epidermal cells. Appressoria are swollen structures from which thin penetration tubes grow into plant cell walls. Intracellular hyphae produce clusters of rounded thick-walled cells within cortical cells, called microsclerotia, which may serve a storage function.

DSE fungi can break down various organic substances including cellulose, starch, xylan, and gelatine by secreting enzymes such as laccases, lipases, amylases and polyphenol oxidases (Jumpponen & Trappe, 1998). The interaction between DSE fungi and their host occurs along the mutualism-parasitism continuum. As with AM, positive, neutral and negative effects on plant biomass have been documented, which may depend on the plant-fungus combinations and environmental conditions (Newsham, 1999; Newsham, 2011; Jumpponen, 2001; Tellenbach, Grünig & Sieber, 2011). DSE also protect their hosts against plant pathogens, herbivores, and abiotic stresses such as heat that could be linked to their capability of synthesizing antibacterial and antifungal compounds, toxic secondary metabolites, or high melanin contents of their hyphae (Mandyam & Jumpponen, 2005).

DSE may also form hyaline (non-melanized) structures along with their typical dark septate structures, sometimes referred to as hyaline endophytes (HE) (Haselwandter & Read, 1982; Newsham, 1999; Yu, Nassuth & Peterson, 2001; Lukesova et al., 2015). While axenic cultures of the DSE fungus *Phialocephala fortinii* are generally darkly pigmented, some hyaline thin-walled hyphae were noticed to become thick-walled and melanized on PDA cultures (Currah & Tsuneda, 1993). However, it has also been proposed that HE may be formed by fungal taxa different from the DSE (Vare, Vestberg & Eurola, 1992).

Whether these hyaline septate hyphae are a phase of DSE or an independent entity (HE), they have been underestimated or ignored because their hyaline structures are not usually visible under the ordinary light microscopy and also due to the limitation of staining techniques such as use of Trypan blue. The lipid specific stain Sudan IV and differential interface contrast are required for detection and quantification of HE within roots (Barrow & Aaltonen, 2001; Barrow, 2003).

### Fire-associated mutualism (FAM)

FAM is a recently discovered symbiosis that occurs between cheatgrass (*Bromus tectorum*) and certain isolates of the ascomycetous fungus *Morchella* (Baynes et al., 2011). Fungal hyphae extensively colonize roots in a manner not seen in other mycorrhiza and can further extend towards stem tissues. *Morchella* species may form a weak and probably facultative boitrophic association with plant roots. An EEM-like association (patchy mantle, poorly developed Hartig net, and intracellular colonization) has been observed in pure culture synthesis trials between *Morchella* spp. and *Pinaceae* (Dahlstrom, Smith & Weber, 2000). ECM structures were not detected in synthesis experiments involving other *Morechella* species (Godbout & Fortin, 1985; Warcup, 1990; Yamada & Katsuya, 1995).

As mycorrhizal structures are largely missing in the FAM association, details of root colonization features need to be further investigated. The *Morchella* isolates were shown to significantly increase the biomass of cheatgrass, fecundity of its local population, and also enhance survival rate of seeds exposed to heat. FAM is an evolutionarily novel interaction associated with fire adaptation in which both probability of fire (via increased plant biomass) and plant survival (larger under-ground seed bank) are enhanced, simultaneously (Baynes et al., 2011). Thermotolerance in plants can be possibly linked to production of secondary metabolites (such as monocillin I) by their symbiotic fungal partners (McLellan et al., 2007).

### Feremycorrhiza (FM)

Feremycorrhiza is a plant-fungus symbiosis discovered recently, between jarrah (*Eucalyptus marginata*) and the basidiomycetous fungus *Austroboletus occidentalis* (Kariman et al., 2014a). The term “Feremycorrhiza” originates from “fere” (meaning nearly in Latin and companion in Middle English) and “mycorrhiza”, and refers to basic commonalities shared with mycorrhizal associations in terms of plant growth and nutritional benefits. The potential range of host plants and fungal partners capable of forming FM symbiosis needs to be investigated. A *Scleroderma* species was observed to establish FM or ECM symbiosis with jarrah, depending on the experimental conditions (Kariman et al., 2012; Kariman et al., 2014a; Kariman et al., 2016). This is an example where the symbiosis type (here, presence or absence of fungal interface structures) differs according to environment rather than host. Likewise, Navarro-Ródenas et al. (2012) demonstrated that the type of mycorrhizal association (ECM or EEM) between *Helianthemum almeriense* and *Terfezia claveryi* is determined by the P source where the organic P form can lead to intracellular (EEM) colonization. In FM symbiosis, fungal hyphae live in the rhizosphere soil and vicinity but do not penetrate plant roots. Lack of root colonization was observed microscopically and further confirmed biochemically. Mannitol, a sugar alcohol found in hyphae of *A. occidentalis* and many ECM fungi, was not detected in FM roots whereas it was abundant in ECM roots (colonized by *Scleroderma* sp.), supporting the lack of direct association (Kariman et al., 2014a).

The FM symbiosis can dramatically enhance plant biomass and uptake of nutrients such as N, P, K, S, Mg, Fe, and Zn (Kariman et al., 2012; Kariman et al., 2014a). A study using ^33^P-labelled phosphate revealed that fungal hyphae do not extend far beyond the rhizosphere and do transfer nutrients to the host. The transcript abundances of two plant phosphate transporter (*PHT1*) genes (*EmPHT1* and *EmPHT2*) were not affected in FM roots but were significantly reduced in ECM roots. Enhanced concentration of carboxylates (citrate, in particular) in the rhizosphere of FM plants was correlated with positive nutritional responses. The lack of detectable *PHT1* gene response in FM roots coupled with enhanced carboxylate concentrations in the rhizosphere soil suggests that plant roots function as the active pathway of nutrient uptake in FM symbiosis, i.e., enhanced nutrient solubilization and mobilization seem to be the main functional mechanisms. In ECM symbiosis, however, the reduced concentration of carboxylates in the rhizosphere soil compared to NM (control) plants and down-regulation of two *PHT1* genes in ECM roots (Kariman et al., 2014a) indicates a hyphal pathway as the main route of nutrient acquisition. Improved host performance was observed for FM plants under P-deficiency (Kariman et al., 2012; Kariman et al., 2014a) and phosphate/phosphite toxicity conditions (Kariman et al., 2014b; Kariman et al., 2016). Ability of *Scleroderma* sp. to form FM or ECM associations in the same host (jarrah) depending on the experimental conditions (Kariman et al., 2012; Kariman et al., 2014a; Kariman et al., 2016) suggests a complexity to the detail of the potential fungal-host interactions that deserves further investigation.

In our previous studies, *A.occidentalis* was found to have similar (Kariman et al., 2014a) or superior (Kariman et al., 2012) positive effects on growth of jarrah seedlings compared with the ECM fungus *Scleroderma* sp. Furthermore, both FM and ECM symbioses significantly improved shoot content of P, K, Mg, S, Fe, Zn, and Cu compared to NM jarrah plants (Kariman et al., 2012; Kariman et al., 2014a), whereas improved N nutrition was only observed in the FM symbiosis (Kariman et al., 2014a). Except for N, all these plant nutrients are ultimately derived from weathering of primary minerals in soil. Ectomycorrhizal fungi, mostly basidiomycetes, can mobilize nutrients from insoluble mineral sources (such as apatite, biotite and muscovite) via excretion of carboxylates (low molecular weight organic acid anions) and protons, which results in acidolysis of soil minerals by dismantling their crystalline structure and subsequent release of immobile essential nutrients for plant uptake (Landeweert et al., 2001; Van Scholl, Smits & Hoffland, 2006; Courty et al., 2010). The FM fungus seems to have a remarkable capacity for biological weathering of soil minerals and nutrient mobilization, which was reflected in the significant growth and nutritional benefits for host plants (Kariman et al., 2012; Kariman et al., 2014a; Kariman et al., 2016). Improved P nutrition of jarrah plants associated with the FM fungus could be due to the fungal role in dissolution of phosphate-bearing minerals such as apatite (Newman, 1995; Landeweert et al., 2001; Courty et al., 2010). Feremycorrhiza is an interesting example indicating how an invisible plant-fungus association (no root colonization) can significantly enhance plant fitness, i.e., an important ecological phenomenon that has been missed by traditional structure-focused approaches.

### Recent observations on root endophytes and root-colonizing saprotrophic fungi

Mycobiome studies using the state-of-the-art “omics” technologies (e.g., metagenomics and transcriptomics) have unearthed much more complexity in root and rhizosphere-associated mycobiome than previously presumed. Other than the well-studied root endophytic associations described here (SE and DSE), a multitude of root endophytic and root-colonizing saprotrophic relationships involving diverse fungal lineages have been revealed in root endosphere and rhizosphere of mycorrhizal and NM plants in both natural and farming ecosystems. Similar to SE and DSE, these endophytic associations do not form specialized interface structures in roots (with a few exceptions, see below), but they might possess plant growth-promoting capabilities. However, we should bear in mind that “omics” studies usually do not discriminate between fungal mycelia (active stage) and spores (dormant stage). Accordingly, the “root-colonizing saprotrophs” might be just a biased conclusion of a casual encounter between fungi and plant roots, unless specific microscopic localization probes are employed. *In vitro* and ”omics” studies can reveal the fungal genetic potential for a specific trait, and subsequent research under natural conditions can determine if the identified traits are functional in nature or not, and if so, how these potentials can be exploited to improve or sustain farming/natural systems. Here, we briefly explore several examples of root endophytic/rhizospheric associations discovered recently.

Almario et al. (2017) studied the root-associated mycobiome of the NM plant *Arabis alpina* (*Brassicaceae*) using the amplicon sequencing of the fungal ITS2 region. Fifteen fungal taxa were consistently recovered from roots of *Arabis alpina* plants growing in P-impoverished soils, including a highly abundant Helotiales taxon. The mycobiome associated with roots and rhizosphere of *A. alpina* was dominated by ascomycetes, which is similar to those of the mycorrhizal plants poplar, agave, and plants from an oak-dominated temperate forest (Shakya et al., 2013; Toju, Sato & Tanabe, 2014; Coleman-Derr et al., 2016). The fungal isolate F229 (belonging to Helotiales) showed a mycorrhiza-like activity that included colonization of the root endosphere (inter- and intracellularly) and P transfer to roots. This association improved P uptake and growth of *A. alpina* plants grown in native low-P soil i.e., commensalism. However, potential benefits to the fungal partner have not been investigated. Likewise, the root endophyte *Colletotrichum tofieldiae* transfers P to the NM plant *A. thaliana* and improves its growth only under P-limiting conditions, where the plant growth promotion property of the fungus requires both host plant’s phosphate starvation and innate immune responses (Hiruma et al., 2016). Thus, establishment of this *C. tofieldiae*-*A. thaliana* association seems to somehow depend on P starvation signalling pathway as also observed in AM symbiosis (Karandashov et al., 2004; Chen et al., 2011). Net negative effects of certain root endophytes on growth of *A. thaliana, Microthlaspi erraticum*, and *Hordeum vulgare* have also been documented, which were attributed to incompatibility between symbionts (Kia et al., 2016).

Toju et al. (2013) studied the root mycobiome of 12 plant species from an oak-dominated temperate forest and observed diverse ECM taxa including clades of *Russula*, *Lactarius*, *Cortinarius*, *Tomentella*, *Amanita*, *Boletus*, and *Cenococcum*. Interestingly, the root mycobiome was dominated by endophytic ascomycetes belonging to Helotiales, Chaetothyriales, and Rhytismatales. Similarly, sequencing of the ITS and/or D1-D2 regions of the LSU rDNA using a 454 sequencing platform revealed that endophytic ascomycetes were dominant in root endosphere and rhizosphere mycobiome of *Populus deltoides*, *P. trichocarpa*, *Quercus alba*, and *Pinus taeda* (Bonito et al., 2016). Glynou et al. (2016) recovered 296 operational taxonomic units (OTUs) of root endophytes from roots of the NM plant *Microthlaspi*, which were dominated by six widespread OTUs belonging to the orders Pleosporales, Hypocreales and Helotiales. Here, the local environment was found to be the main factor determining root-endophytic diversity.

Grelet et al. (2017) investigated the associations of *Mycena* species with *Ericaceae* plants, and suggested that *Mycena* species operate along the saprotrophic-symbiotic continuum. A *M. galopus* isolate promoted the growth of *Vaccinium corymbosum* seedlings. The fungus colonizes plant roots and forms distinctive peg-like structures. The positive growth response in plants inoculated with *M. galopus* was similar to that of the plants inoculated with the ERM fungus *Rhizoscyphus ericae*. A shift from saprotrophy towards biotrophy has also been suggested for wood-decaying basidiomycetes. Smith et al. (2017) investigated this phenomenon by using 201 wood-decaying basidiomycetes and monitored their root colonization capacity in two conifers (*Picea abies* and *Pinus sylvestris*). Interestingly, thirty-four fungal species colonized the roots of at least one tree species. Two species formed a mantle around roots and one species (*Phellinus igniarius*) formed Hartig net-like structures. These typical endophytic associations between wood-decaying fungi and roots of ECM hosts have been proposed to be an intermediary step between saprotrophic and biotrophic (mycorrhizal) strategies (Selosse, Dubious & Alvarez, 2009; Baldrian & Kohout, 2017). Here, the emerged endophytic behavior of these fungi could be driven by easily available C source from roots, which is rendered inaccessible to competing saprotrophs by active plant defense pathways (Baldrian & Kohout, 2017). Hacquard et al. (2016) compared the transcriptomes of the beneficial root endophyte *C olletotrichum tofieldiae* and its pathogenic relative *Colletotrichum incanum*, and found genomic signatures dealing with transition from pathogenic to beneficial lifestyles including a narrowed repertoire of secreted effector proteins, and expanded families of genes related to chitin binding and secondary metabolism.

Mycobiome studies have unearthed another class of fungal lineages i.e., Archaeorhizomycetes that occurs ubiquitously in different terrestrial ecosystems. These fungi have saprotrophic potential, occupy roots and rhizosphere soil, but do not form recognizable mycorrhizal structures (Schadt et al., 2003; Rosling et al., 2011). Due to their dominance in summer and absence during other times of the year, Archaeorhizomycetes have been suggested to be dependent on root-derived C compounds as the main C source (Schadt et al., 2003). Several members of Archaeorhizomycetes have been cultured *in vitro* (Rosling et al., 2011), which can facilitate studying their functioning, that is rather vague currently.

All these examples indicate that root-associated fungi and free-living saprotrophic fungi may have evolved the capacity to form facultative biotrophic or mycorrhiza-like associations, which could enhance plant phenotype as reported for *Mycena galopus* (Grelet et al., 2017). Development of peg-like structures in *Mycena* species (Grelet et al., 2017), Hartig net-like structures in *Phellinus igniarius* (Smith et al., 2017), and formation of arbuscule-like structures in FRE belonging to Mucoromycotina (Orchard et al., 2017) suggest that mycorrhiza and mycorrhiza-like associations might have evolved in numerous fungal lineages independently.

### Root-fungal symbioses and ecosystem functioning

Mycorrhizas and other fungal root symbionts are involved in key ecosystem processes including mineralization of organic matter, biological weathering of soil minerals, solubilization of mineral nutrients, soil acidification, C cycling, interactions with myco-heterotrophic plants, mediation of plant responses to biotic and abiotic stresses, tolerance to heavy metals, shaping plant and microbial communities, and enhancing biodiversity (Landeweert et al., 2001; Finlay, 2008; Van der Heijden, Bardgett & Straalen, 2008). Most studies dealing with root-fungal interactions have focused on plant performance at the individual level, and the ecosystem-scale effects warrant further and more in-depth investigations. AM and ECM associations seem to be key players in this regard, while certain less-explored associations (e.g., complex endophytic mycobiomes, FE, and DSE) could also play a major role in ecosystem functioning due to their ubiquitous presence across various ecosystems.

Different associations may function differently in ecosystem processes. A typical example for differential impact of root-fungal symbioses on a key ecosystem process has been presented by Phillips, Midgley & Brozstek (2013), who classified the temperate forests based on their mycorrhizal associations of the dominant trees and their contrasting effects on the nutrient economy. Their framework suggested that forests dominated by AM trees have an inorganic nutrient economy, in which plant-derived C is rapidly decomposed by saprotrophs followed by rapid cycling of the inorganic nutrients. However, forests dominated by ECM trees have an organic nutrient economy, due to slow turnover of plant-derived C and enhanced root/rhizosphere couplings, leading to higher availability of organic nutrients.

Soil microbes (including fungal root symbionts) and N availability are among the main factors controlling decomposition of organic matter in soil (Allison et al., 2010; Lindahl, De Boer & Finlay, 2010). ECM and ERM fungi have greater access to organic N sources than AM fungi, due to possession of N-degrading enzymes and proteases (Read & Perez-Moreno, 2003), suggesting that soil C storage is greater in ecosystems dominated by EEM fungi compared with those dominated by AM fungi (Averill, Turner & Finzi, 2014).

Soil microorganisms, mycorrhizas in particular, are the key factors determining P cycling in forest soil ecosystems, rather than the annual uptake of trees. Rosling et al. (2016) uncovered significant differences in P cycling and mycorrhizal functioning in plots dominated by AM and ECM-associated trees in hardwood forests. Their results showed higher phosphatase activities and a larger organic P pool in ECM plots than in AM plots, while inorganic P decreased and organic P increased over the growing season in both ECM and AM plots. Interestingly, a similar microbial biomass (including symbiotic and saprotrophic microbiota) was observed for both AM and ECM plots and the microbial P pool was almost three times larger than the annual P uptake by the existing vegetation. Although this large microbial biomass can potentially exacerbate P-limitation for plant growth, these forests are still productive based on the annual litter fall datasets (Rosling et al., 2016). This is possibly because the soil available P pool is being continuously topped up mainly through mobilization of phosphate-bearing minerals, rather than mineralization of organic P compounds in soil that may not meet both microbial and plant P demands.

These examples suggest that functional traits, trophic habits and enzymatic properties of different mycorrhizal types determine their role and significance in key ecosystem processes such as N, P, and C cycling. These studies provide useful models for measuring the impact of other less-studied root-fungal symbioses at an ecosystem scale. The studies mentioned above have primarily focused on nutrient cycling, while these frameworks/models can be potentially employed to determine fungal effects on plant communities subjected to environmental constraints within diverse ecosystems. For instance, FRE are ubiquitous in harsh environments (Crush, 1973; Postma, Olsson & Falkengren-Grerup, 2007; Newsham, Upson & Read, 2009; Orchard et al., 2016), and it would be interesting to explore their role in functionality of stressful ecosystems such as those with high altitude, soil acidity or cold temperatures.

## Conclusions

Our review of the current literature suggests that the potential exchange of nutrients/metabolites and beneficial interactions in plant-fungal relationships can occur via specialized interface structures or simple non-specialized hyphae inside or outside roots. This fundamental fact has been either missed or neglected (due to invisibility of certain symbioses or sole focus on specialized structures), but it is essential for a proper understanding of the actual contribution of fungal root symbionts to nutrient cycling and plant fitness in terrestrial ecosystems. Nevertheless, we do emphasize that while interface morphological characteristics are not a direct indicator or prerequisite for enhancing plant phenotype, certain highly efficient structures (such as those in AM and ECM associations) are key elements for mycorrhizal functionality at both plant and ecosystem levels. Overall, interface structures can be morphologically diverse, specialized (arbuscules, Hartig nets and fungal pegs: AM, ECM, EEM, ABM, MTM and FRE), non-specialized (hyphae and hyphal coils: ERM, SERM, OM, DSE and SE), or totally absent (FM) in root-fungal relationships, suggesting plasticity in interface structures as well as functional traits of root-associated fungi i.e., employing different strategies such as direct nutrient transfer and/or rhizosphere modification to benefit their host plants. To shed more light on the nature of a given root-fungal relationship including some currently known associations, it is crucial to explore mutualism (i.e., a reciprocal exchange of nutrients/metabolites) as the definitive feature in mycorrhizal symbiosis.

Specialized structures such as arbuscules could be a more efficient means of nutrient transfer due to their specific features such as increased intimate surface area at the symbiotic interface. To date, most studies have focused on AM, ECM, OM and SE, and further research is required to unearth the cellular mechanisms behind exchange of nutrients in the other root-fungal interactions. Sequencing the genome of representative fungi will identify the potential trophic habits of the fungal partner, and, in a given association, the symbiotic characteristics can be explored via transcriptomic analyses whilst proteomic studies focusing on nutrient/hexose transporters at the interfacial matrix will explore further the symbiotic benefits. Comparative genomics of the fungal symbionts and their hosts would provide key insights into their life-style and evolution.

State-of-the-art molecular approaches have unearthed much more complexity and diversity in root-fungal relationships than previously thought, and suggest that mycorrhizal capacity (or saprotrophy to biotrophy transition) has possibly evolved in highly diverse fungal lineages across the kingdom Mycota. This includes the recent discoveries of diverse root endophytes and root-colonizing saprotrophic fungi, some with peg-like (*Mycena galopus*), Hartig net-like (*Phellinus igniarius*), or arbuscule-like (FRE belonging to Mucoromycotina) structures, which could possibly be of a mutualistic nature. Moreover, dual niches for mycorrhizal and root endophytic fungi seems to be a common strategy, and could be a key capacity, aiding them to cope with environmental constraints and ecological restrictions.

## Additional Information and Declarations

### Data Availability

The following information was supplied regarding data availability: This is a review paper and the research in this article did not generate any data or code.
